# The protective effect of the EP2 receptor on TGF-β1 induced podocyte injury via the PI3K / Akt signaling pathway

**DOI:** 10.1371/journal.pone.0197158

**Published:** 2018-05-10

**Authors:** Jing Liu, Yi-de Zhang, Xiao-lan Chen, Xue-ling Zhu, Xu Chen, Jian-hua Wu, Nai-feng Guo

**Affiliations:** Department of Nephrology, the Affiliated Hospital of Nantong University, Nantong, China; Hopital Tenon, FRANCE

## Abstract

Transforming growth factor β1 (TGF-β1) plays a central role in chronic kidney diseases. TGF-β1 induction causes podocyte injury, which results in proteinuria and renal failure. However, the effect of the prostaglandin E2 /E-prostanoid receptor (EP2) on TGF-β1-induced podocyte injury remains unknown. Previous studies have shown that phosphoinositide 3-OH kinase (PI3K)/Akt is widespread in cells, and is vital for the regulation of cell proliferation, differentiation, apoptosis and metabolism. In this study, we cultured immortalized mouse podocytes in vitro in different groups: control group; TGF-β1 (5ng/ml) group; EP2 agonist Butaprost treatment (10^−7^, 10^−6^, or 10^-5^mol/L) +TGF-β1 group; EP2 antagonist AH6809 treatment (10^−7^, 10^−6^, or 10^-5^mol / L) + TGF-β1 group. We found that compared with the control group, proliferation of podocytes in the TGF-β1 group significantly decreased and apoptosis increased. Expression of cAMP decreased, whereas PGE2 increased. Meanwhile, expressions of nephrin, podocin and CD2AP mRNA and protein were dramatically downregulated, activated caspase-3 was increased, and activated PI3K/Akt activity were depressed. Butaprost intervention promoted podocyte proliferation with reduced apoptosis. Conversely, AH6809 intervention led to opposite results (P<0.05). Our findings suggested that EP2 agonist protects podocytes by increasing expression of cAMP, which creates feedback of inhibiting PGE2 expression. This causes the interaction of nephrin, podocin and CD2AP resulting the inhibition of apoptosis induced by activation of the PI3K / Akt signaling pathway.

## Introduction

The podocyte is a terminally differentiated epithelial cell, they adhere at the surface of glomerular basement membrane(GBM), stretch foot processes, and intercross to form slit diaphragms(SDs) on GBM, forming an important component of glomerular filtration barrier(GFB)[1]. Previous studies have found many protein components of SDs, including nephrin, podocin, CD2AP, ZO-1, P-cadherin, FAT and Neph1. All of these molecules construct the “SD complex” which participates in maintaining cytobiologic functions such as proliferation, differentiation, survival, endocytosis and construction of cytoskeleton [2, 3]. The principal structural component of SD is a large molecular weight zipper-like protein known as nephrin. Nephrin is an immunoglobulin-type cell adhesion molecule that is critical to the SD function. In addition, nephrin acts as an anchor for actin filaments, which subserve the contractile function of the podocyte via connection with synaptopodin. Previous studies showed that a tyrosine residue in the nephrin cytoplasmic region could be phosphorylated by Src family kinase Fyn, resulting in interaction between nephrin and podocin, as well as downstream signal pathway enhancement [4]. Podocin has a hairpin-like structure and embeds in the membrane, interacts with nephrin and CD2AP via cytoplasmic carboxyl terminus, and then mediates connection between SD and podocyte cytoskeleton to stabilize podocytes[5]. CD2AP is located on the podocyte cytoplasmic side, it can not only adjust cytoskeleton arrangement by connecting directly with F-actin, but also conduct signal transduction by combining directly with nephrin and podocin. In this regard, CD2AP is one of the most important components in maintaining the normal ultra-structure and function of SD [6].

Phosphatidylinositol-3-kinase (PI3K) family serves as a second messenger related to intracellular signal transduction. Akt is activated by PI3K, and the PI3K/Akt signaling pathway plays a critical role in (the) resistance of podocyte apoptosis. Podocyte injury induced by purine adenosine, AngⅡ, TGF-β1, protein overload, hemodynamic disorder, etc. can give rise to proteinuria[7]. Studies have shown that podocyte injury may affect progression of diseases by intervening with the target of PGE_2_ receptor subtype [8]. PGE_2_ is a cardinal metabolic product of arachidonic acid, and generally participates in various kidney physiologic and pathophysiologic processes, through interacting with four subtypes of prostaglandin receptors coupled with G protein[9]. Four different EP receptors belong to the G protein coupled receptor family, but they have different G protein coupling selectivity. EP2 mainly couples with Gs protein, enhances intracellular cAMP level, and activates PKA and its downstream signaling molecules. Our previous study showed that EP2 overexpression in models of both TGF-β1 induced mesangial cell injury and 5/6 nephrectomy mice could ameliorate mesangial cell proliferation and progression of kidney fibrosis progression [10].

However, the concrete mechanism of EP2 on protecting podocytes from injury has not been fully elucidated to date. The present study investigated the function and mechanism of EP2 on TGF-β1 induced podocyte injury and apoptosis, SD protein expression, as well as PI3K/Akt signaling pathway by treating podocytes with different doses of EP2 agonist and antagonist.

## Materials and methods

### Materials and reagents

Butaprost and AH6809 were purchased from Caymen (USA), Recombinant human TGF-β1 was purchased from PeproTech (UK), The CCK-8 kit and Trizol RNA extracting kit were purchased from Invitrogen (USA). Mouse cAMP and PGE2 ELISA kit were purchased from Weston Biology Company (Shanghai, China). The reverse transcription (RT) kit was purchased from Fermentas (USA). The real-time quantitative PCR (RT-qPCR) kit was from Roche (USA). The Annexin V-FITC cell apoptosis assay kit was purchased from Beyotime (Shanghai, China). Nephrin, podocin, and CD2AP primers were purchased from Invitrogen (USA). Rabbit anti-mouse polyclonal antibodies for nephrin, podocin and caspase3 were purchased from Abcam (UK). Rabbit anti-rat monoclonal antibodies of GAPDH, CD2AP, PI3K-p85 and Akt were purchased from Cell Signaling (USA), as well as phosphorylated PI3K-p85 and Akt monoclonal antibodies. The horseradish peroxidase (HRP) labeled goat anti-mouse and goat anti-rabbit IgG secondary antibody were purchased from SantaCruz (USA). Alexa Fluor 488 labeled goat anti-rabbit IgG (H+L) was purchased from Fcmacs (Nanjing, Jiangsu province, China).

### Cell culture and grouping

MPC5 mouse podocytes was kindly provided by Professor Peter Mundel at Mount Sinai medical school, New York City, NY, United States. MPC5 podocytes were cultured as previously described [11]. Generation 9~10 podocytes were divided into following groups: ① control group; ② TGF-β1 (5ng/ml) group; ③-⑤ EP2 agonist Butaprost treatment group (10^−7^,10^−6^,10^-5^mol/L) +TGF-β1, respectively; ⑥-⑧ EP2 antagonist AH6809 treatment group (10^−7^,10^−6^,10^-5^mol / L) + TGF-β1, respectively.

### Indirect immunofluorescence staining

Generation 9~10 podocytes were plated in each well of a 24-well tissue culture plate with a glass coverslip. When 40% confluent, the coverslip was processed by fixating, permeating, and blockading. Each coverslip was incubated with primary antibodies against nephrin (1:500) and podocin (1:500) and incubated at 4°C overnight. The coverslips were then washed three times with PBS and incubated with Alexa Fluor 488 (1:100) for 45 minutes at $°C. Finally, cells were observed using a confocal laser scanning microscope (Olympus, FV 1000).

### CCK-8 cell proliferation assay

Cells were pretreated with different concentrations of Butaprost and AH6809 and treated with 5ng/ml of TGF-β1 for 0, 4, 8, 12, 24 h, except for control group. Every well including the control group was treated with 10μl CCK-8 solution and then placed in an incubator for 2 h. Finally, the optical density (OD) value was measured using ELIASA at 450 nm.

### Enzyme—Linked immunosorbent assay

ELISA was used to measure intracellular cAMP and PGE2 according to the manufacture’s instruction.

### RT-qPCR was performed to detect podocyte mRNA expression

Total RNA was extracted from podocytes using the Trizol method and then RT was performed using a high capacity cDNA RT kit according to the manufacture’s instruction. Relative quantification of gene expression was performed using glyceraldehydes-3-phosphate dehydrogenase (GAPDH) as an internal control. (Table 1). Two-step RT-qPCR analysis for the expression of nephrin, podocin and CD2AP was performed using Applied Bio systems Power SYBR-Green PCR Master Mix (USA) in a total volume of 20μl. Reactions were carried out using a RT-qPCR system for 40 cycles (95°C for 15s, 60°C for 5s, 72°C for 30s) after an initial 10 min incubation at 95°C. Data analysis was performed according to the ΔΔCt method.

**Table 1:** Primers used for RT-qPCR.

Gene	sequence (5’-3’)
GADPH	forward AGGAGCGAGACCCCACTAACA
	reverse AGGGGGGCTAAGCAGTTGGT
Nephrin	forward CCCCAACATCGACTTCACTT
	reverse GGCAGGACATCCATGTAGAG
Podocin	forward GCGAGTGGCTTCTTGTCCTC
	reverse AGGTGTCCAGGCAGGGTAGA
CD2AP	forward CCGAGTTGGGGAAATCATCAG
	reverse GTCCATAAGTGCTGATTCGCTG

### Western blot analysis

Western blot analysis was used for detecting expression of podocyte associated proteins. Total proteins were extracted from podocytes using immunoprecipitation cell lysis buffer, and were measured according using an ultraviolet spectrophotometer (UV-1601). Samples were boiled for 5 min, separated using sodium dodecyl sulfate-polyacrylamide gel electrophoresis (SDS-PAGE), transferred to polyscreen membranes (PVDF; Millipore), then blocked at room temperature for 2 h with 5% BSA. The membrane was washed with Tris-Buffered Saline with 0.05% Tween (TBST) for 10 min × 3. The PVDF membrane was incubated with primary polyclonal antibodies (rabbit anti-mouse nephrin, podocin or caspase-3), primary monoclonal antibodies (rabbit anti-mouse CD2AP, PI3K-p58, Akt), or primary phosphorylated monoclonal antibodies (rabbit anti-mouse PI3K-p58 or Akt) (1:1000) at 4°C overnight. The membrane was washed with TBST 3 times followed by incubation with a HRP labeled secondary antibody (goat anti-rabbit) (1:1000) at room temperature for 2 h. The reaction was visualized using the ECL (enhanced chemiluminescence) detection system on the BIO-RAD ChemiDoc XRS. The results were analyzed using Image J software.

### Flow cytometry assay

The cell monolayers were trypsinized and resuspended in the corresponding medium, centrifuged at 3000 rpm for 3 min at 4°C, washed once with cold PBS, and resuspended in annexin V-FITC/PI according to the manufacturer’s recommendation. Flow cytometry was conducted in the UVA Flow Cytometry Core Facility. With annexin V/PI staining, viable cells are annexin and PI negative. Cells that are in early apoptosis are annexin positive and PI negative. However, cells that are in late apoptosis are annexin and PI positive. Necrotic and dead cells are annexin V negative and PI positive.

### Statistical analysis

All experiments were performed in triplicate and the results were expressed as means ± standard deviation for each group. All data were analyzed with SPSS 19.0 software. One-way analysis of variance (ANOVA) was used to determine the level of significance among the groups followed by an alternatively determined by a paired t-test where appropriate. Significant differences were tested using Bonferroni’s post hoc test for multiple comparison. Differences were considered significant when *P*<0.05.

## Results

### Expression of nephrin and podocin protein in podocytes

**Fig 1:**
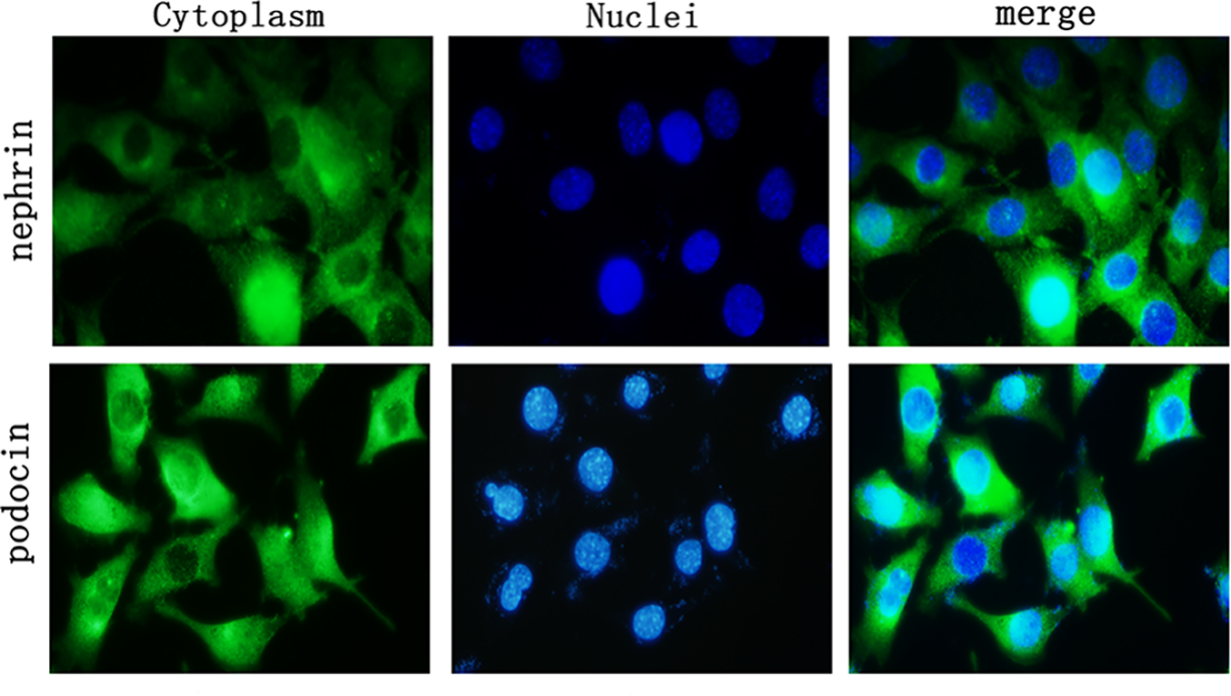
Expression of nephrin and podocin protein in podocytes. Indirect immunofluorescence staining was used to identify podocytes. Podocyte marker proteins nephrin and podocin stained positive, showing green fluorescence; the nuclei were stained with dark blue fluorescence. (400×).

Podocyte marker protein nephrin and podocin were expressed in the cytoplasm of podocytes using indirect immunofluorescence staining (Fig 1).

### Effect of Butaprost and AH6809 on podocytes proliferation induced by TGF-β1

**Fig 2:**
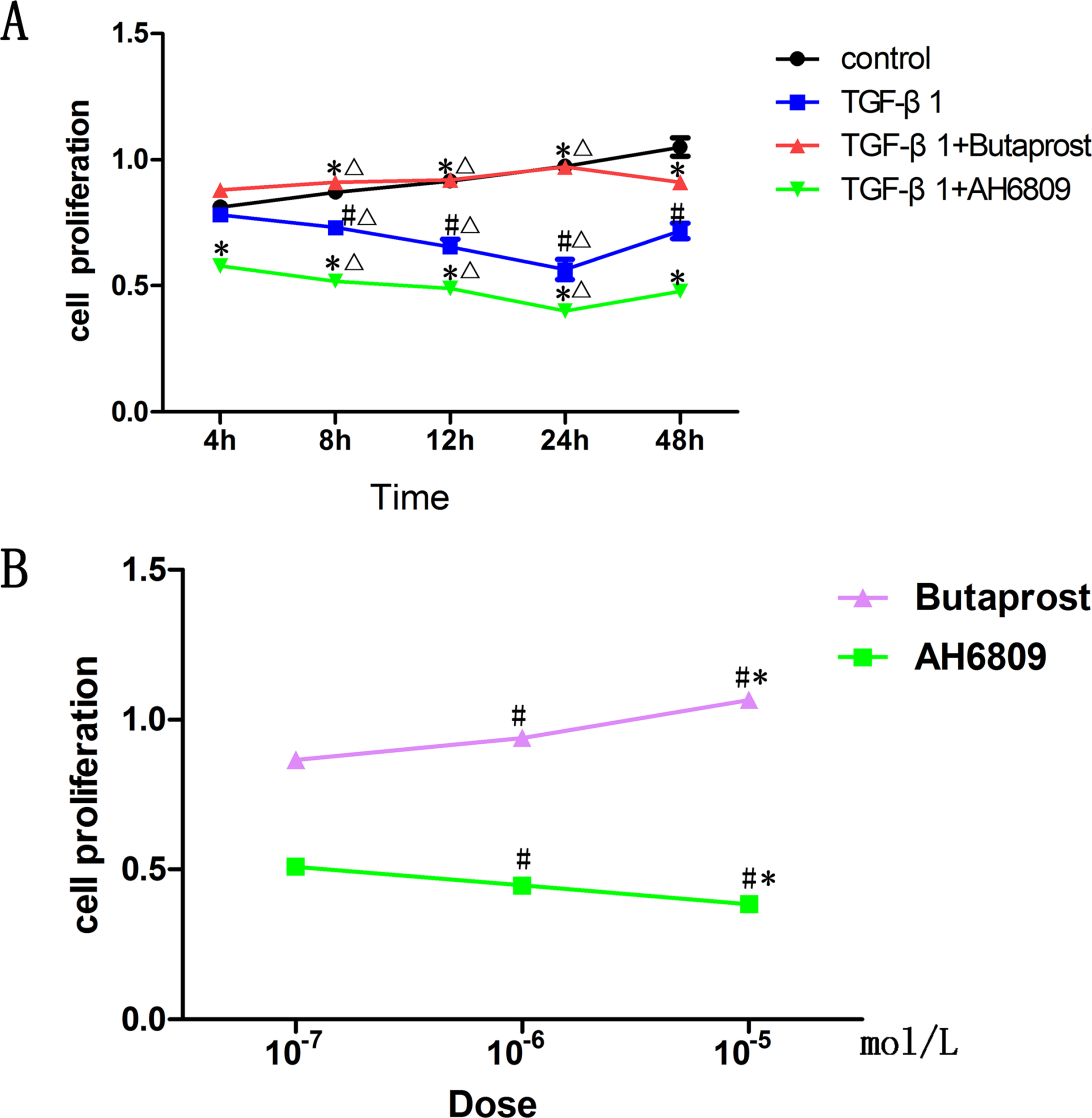
Determination of podocyte proliferation. (A) Podocytes were pretreated with the same concentration (10^-5^mol/L) of Butaprost or AH6809 for 30 min. Thereafter, every well except for control group was treated with 5ng/ml TGF-β1 for 0, 4, 8, 12, 24h (n = 6 each). ^#^P<0.05 compared to control group at the same time point; *P<0.05 compared to the TGF-β1 group at the same time point, ^Δ^P<0.05 compared to the same group at 4 h. (B) Podocytes were pretreated 30 min with different concentrations (10^−7^, 10^−6^, 10^-5^mol/L) [12, 13] of Butaprost or AH6809 (n = 3 each dose). Every well except for the control group was treated with 5ng/ml of TGF-β1 for 24 hours, ^#^P<0.05 compared to 10^-7^mol/L concentration group, *P<0.05 compared to 10^-6^mol/L concentration group.

Cell proliferation rates were assessed using the CCK-8 assay. Cell proliferation in TGF-β1 (5ng/ml) group markedly decreased (*P* < 0.05), and could be further suppressed in a time-and dose-dependent manner by AH6809. In contrast, it could be enhanced in a time-and dose-dependent manner by Butaprost treatment (*P* < 0.05). The effects of both Butaprost and AH6809 gradually increased between 0 and 24 h, reached its peak at 24 h, then began to fade gradually (Fig 2).

### Effect of Butaprost and AH6809 on the level of cAMP and PGE2 in podocyte induced by TGF-β1

**Fig 3:**
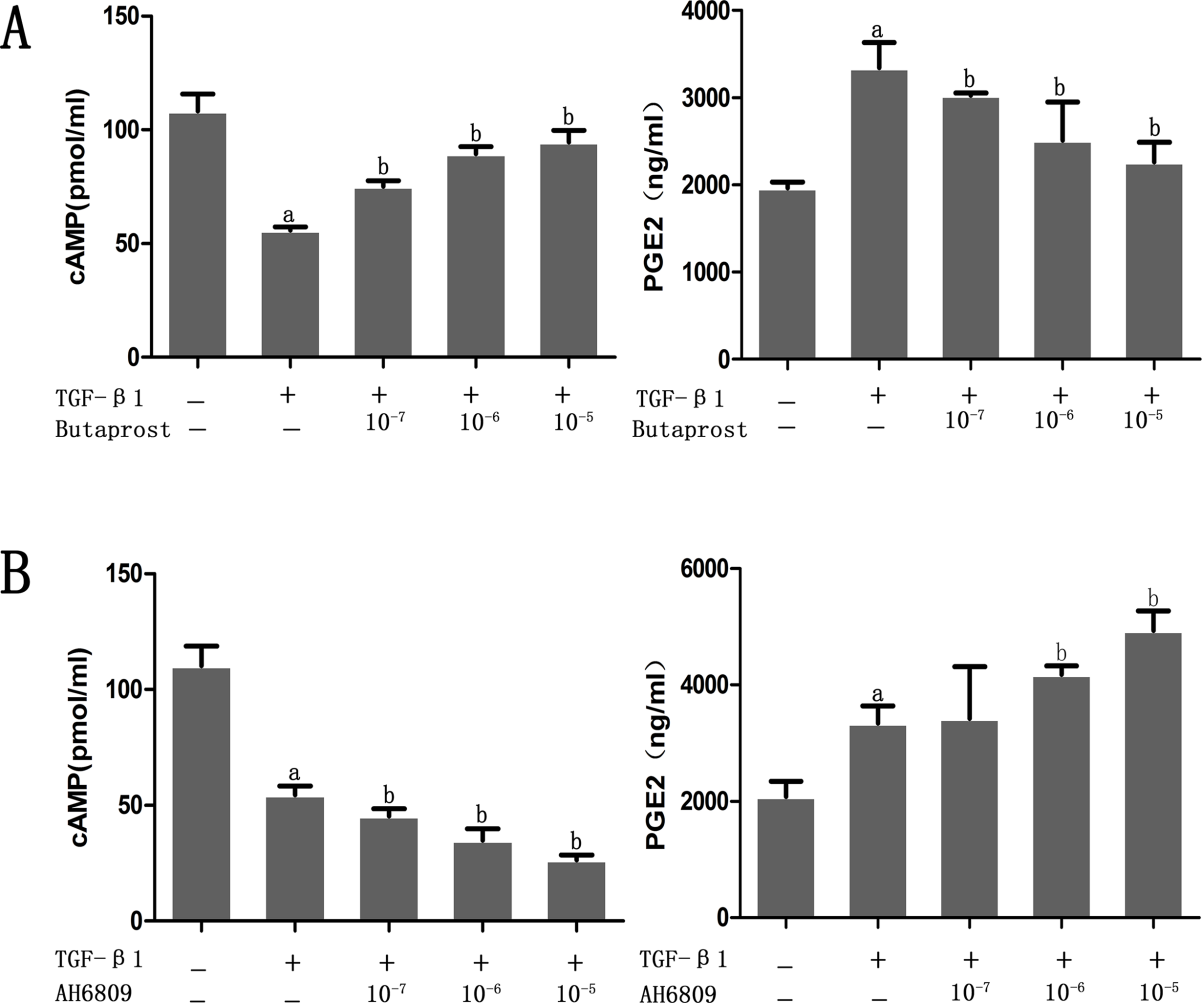
Concentration of cAMP and prostaglandin E2. (A) Podocytes were pretreated with different concentrations (10^−7^, 10^−6^, 10^-5^mol/L) of Butaprost for 30 min (n = 4 each dose). (B) Podocytes were pretreated with different concentrations (10^−7^, 10^−6^, 10^-5^mol/L) of AH6809 for 30 min (n = 4 each dose). Then, every well except for the control group was treated with TGF-β1 (5ng/ml) for 24 h. ^a^P<0.05 compared to the control group, ^b^P<0.05 compared to the TGF-β1 group.

According to the ELISA results, expression of cAMP decreased and PGE2 increased in the TGF-β1 group when compared with the control group. Interestingly, expression of cAMP increased and PGE2 decreased significantly in the Butaprost group compared with the TGF-β1 group. However, expression of cAMP further decreased and PGE2 increased in a dose-dependent manner in the AH6809 group in comparison with TGF-β1 group (all *P* < 0.05) (Fig 3).

### Effect of Butaprost and AH6809 on expression of nephrin, podocin, and CD2AP proteins in podocytes treated with TGF-β1

**Fig 4:**
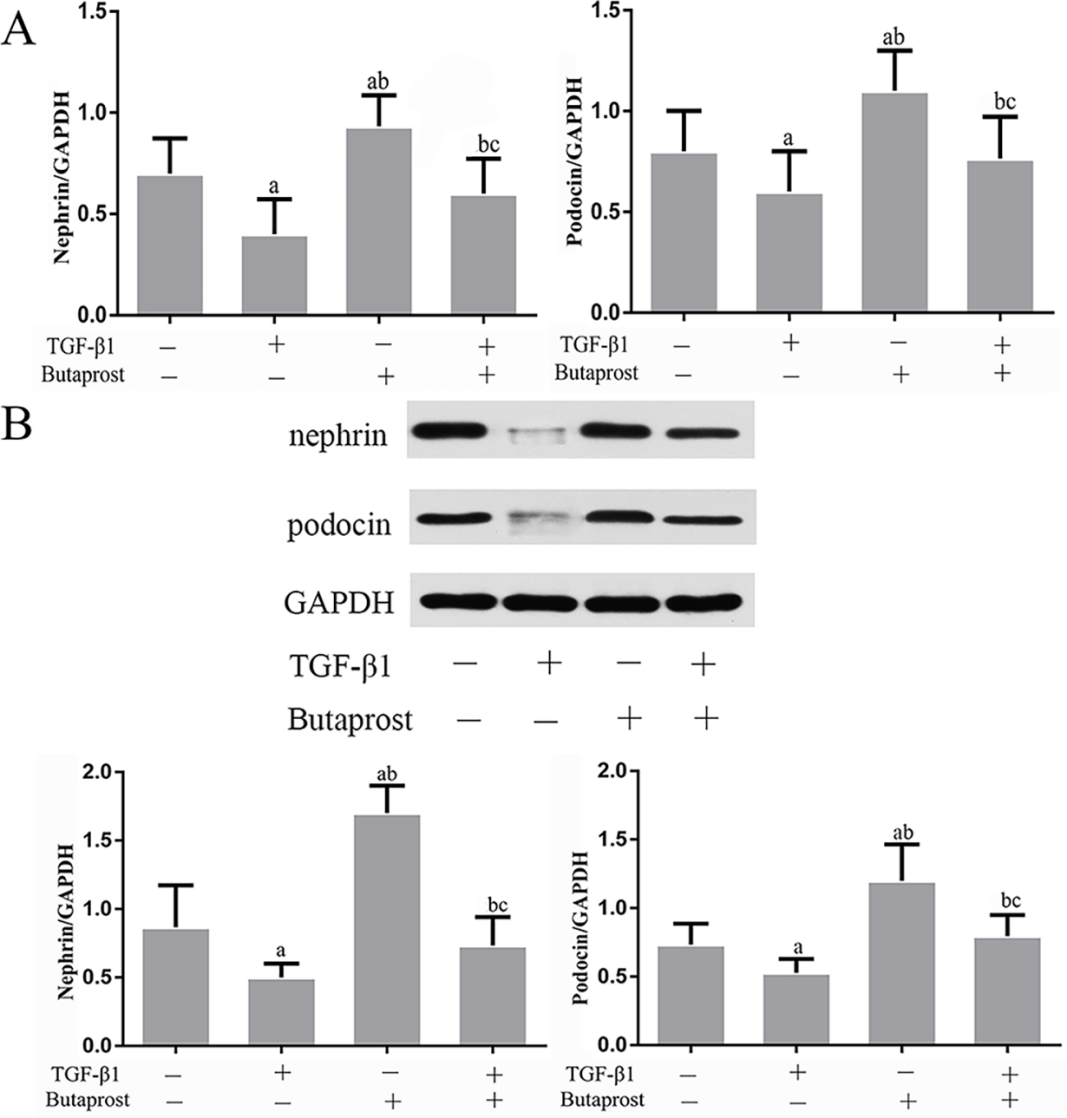
The effect of Butaprost on nephrin and podocin expression in TGF-β1 treated podocytes. Podocytes were pretreated with 10^-5^mol/L of Butaprost for 30 min and then treated with 5 ng/ml TGF-β1 for 24 h. Nephrin and podocin were determined at the mRNA and protein level (n = 6 each).(A:RT-qPCR, B: Western blot). ^a^P<0.05 compared to the control group, ^b^P<0.05 compared to the TGF-β1 group. ^c^P<0.05 compared to the control+ Butaprost group.

**Fig 5:**
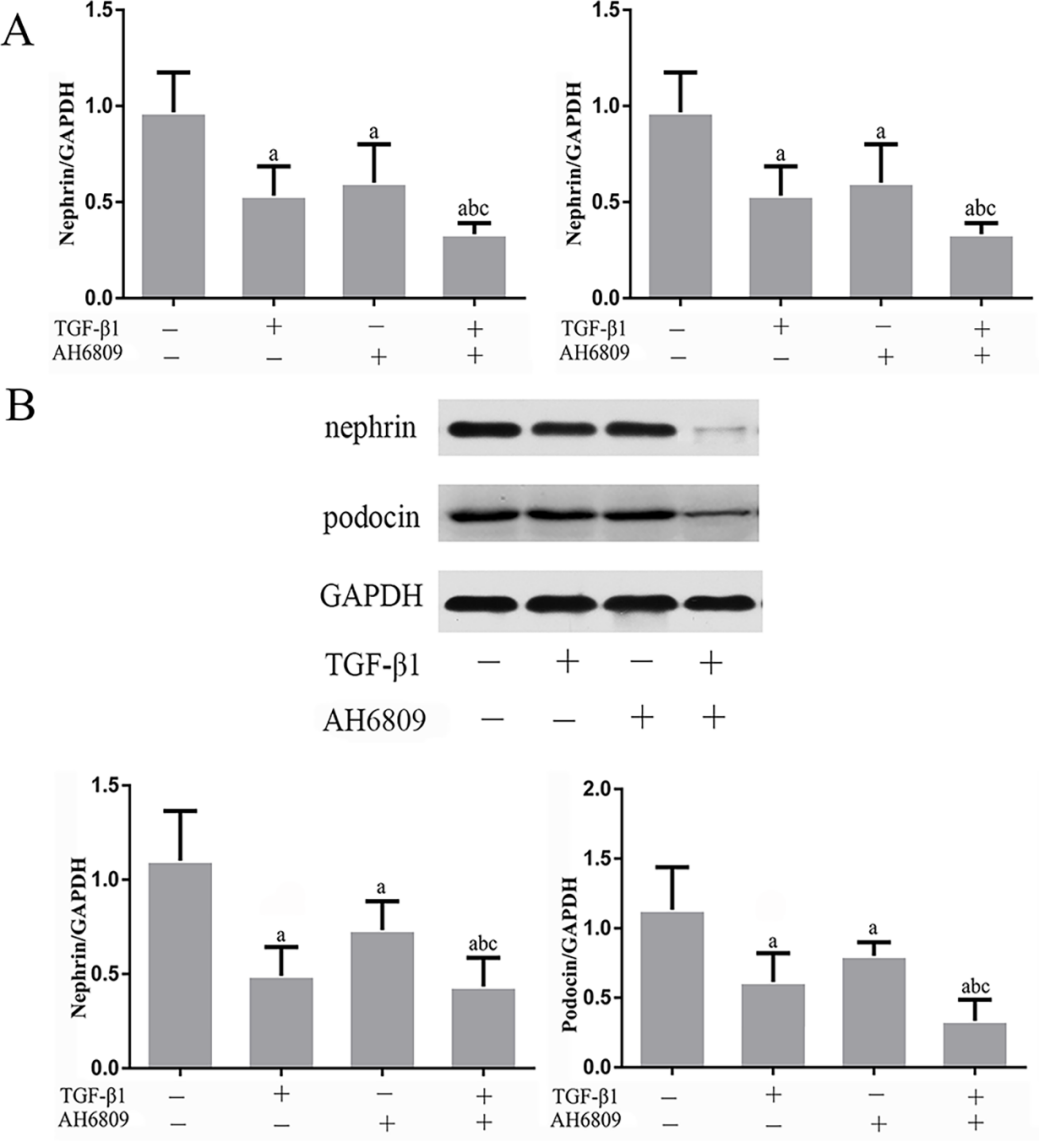
The effect of AH6809 on nephrin and podocin expression in TGF-β1 treated podocytes. Podocytes were pretreated with 10^-5^mol/L AH6809 for 30 min and treated with 5 ng/ml TGF-β1 for 24 h. Nephrin and podocin were determined at the mRNA and protein level (n = 6 each).(A:RT-qPCR, B: Western blot). ^a^P<0.05 compared to the control group, ^b^P<0.05 compared to the TGF-β1 group. ^c^P<0.05 compared to the control+AH6809 group.

**Fig 6:**
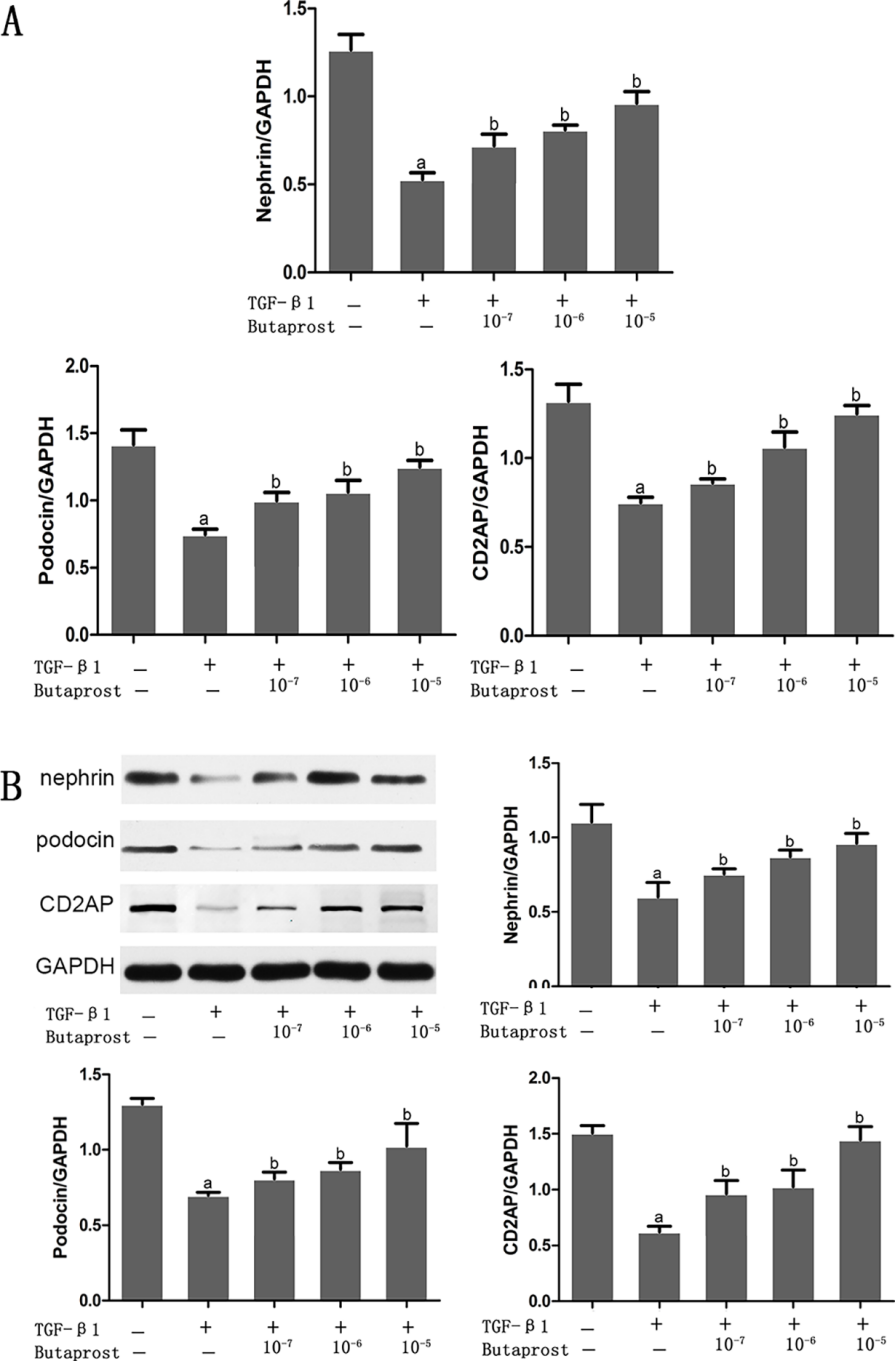
The concentration dependent effect of Butaprost on nephrin, podocin, and CD2AP expression in TGF-β1 treated podocytes. Podocytes were pretreated with different concentrations (10^−7^,10^−6^ or 10^-5^mol/L) of Butaprost (n = 4 each dose) for 30 min and were treated with 5 ng/ml TGF-β1 for 24 h. Nephrin, podocin and CD2AP were determined at the mRNA and protein level.(A:RT-qPCR, B: Western blot). ^a^P<0.05 compared to the control group, ^b^P<0.05 compared to the TGF-β1 group.

**Fig 7:**
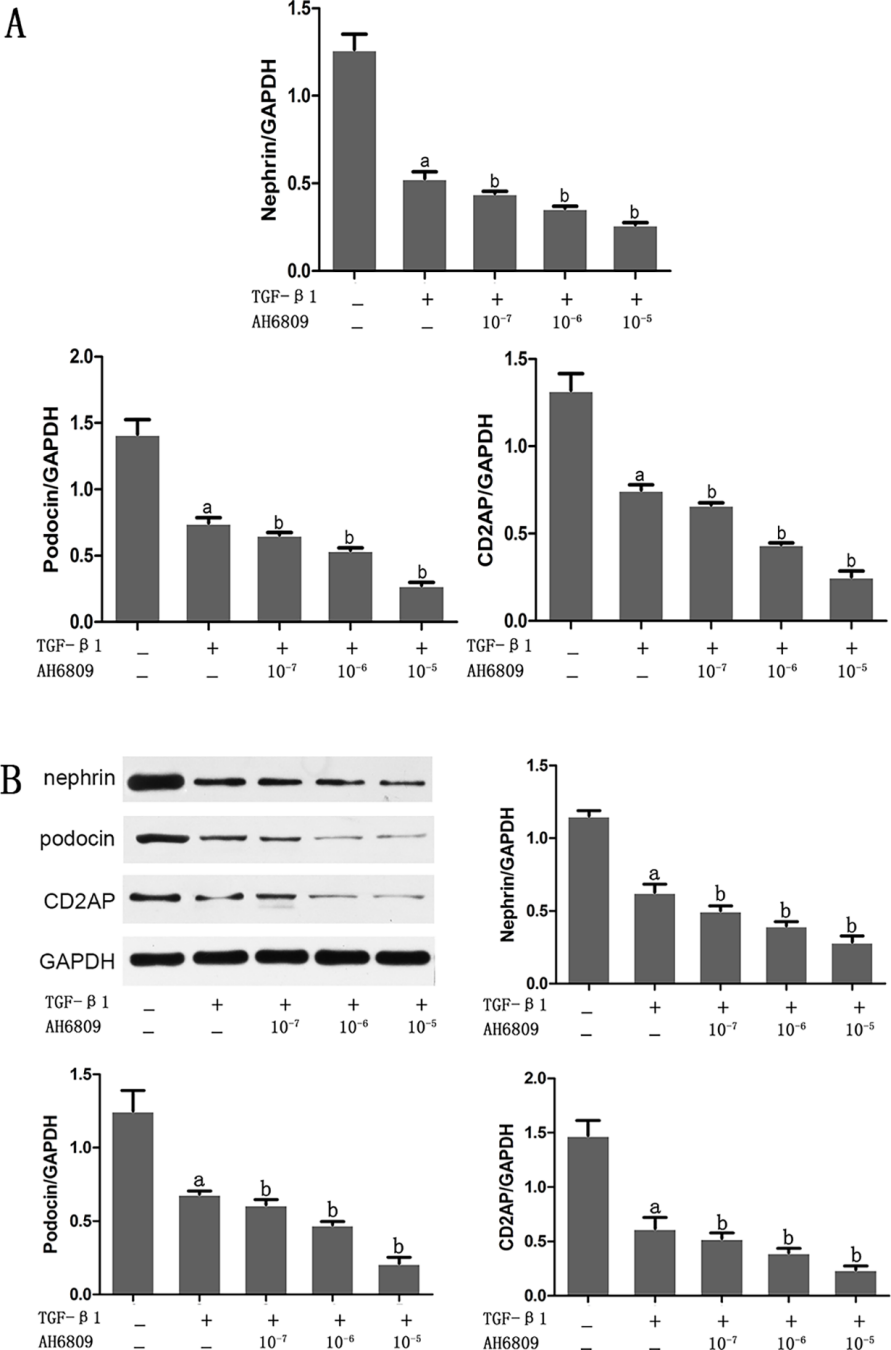
The concentration dependent effect of AH6809 on nephrin, podocin, and CD2AP expression in TGF-β1 treated podocytes. Podocytes were pretreated for 30 min with different concentrations (10^−7^,10^−6^ or 10^-5^mol/L) of AH6809 (n = 4 each dose), and were stimulated with 5 ng/ml TGF-β1 for 24 h. Nephrin, podocin, and CD2AP were determined at the mRNA and protein level.(A:RT-qPCR, B: Western blot). ^a^P<0.05 compared to the control group, ^b^P<0.05 compared to the TGF-β1 group.

Based on real-time fluorescent PCR and western blot results, both mRNA and protein expression of nephrin, podocin and CD2AP prominently decreased in the TGF-β1 group compared with the control group (*P* < 0.05), (Figs 3–7). In comparison with the TGF-β1 group, expression of both mRNA and protein of nephrin, podocin and CD2AP increased in the Butaprost group (Figs 4 and 6), but decreased in the AH6809 group (Figs 5 and 7), both in a time-and dose-dependent manner (*P* < 0.05).

### Effect of Butaprost and AH6809 on expression of activated Caspase-3, PI3K and AKT proteins in podocytes stimulated with TGF-β1

**Fig 8:**
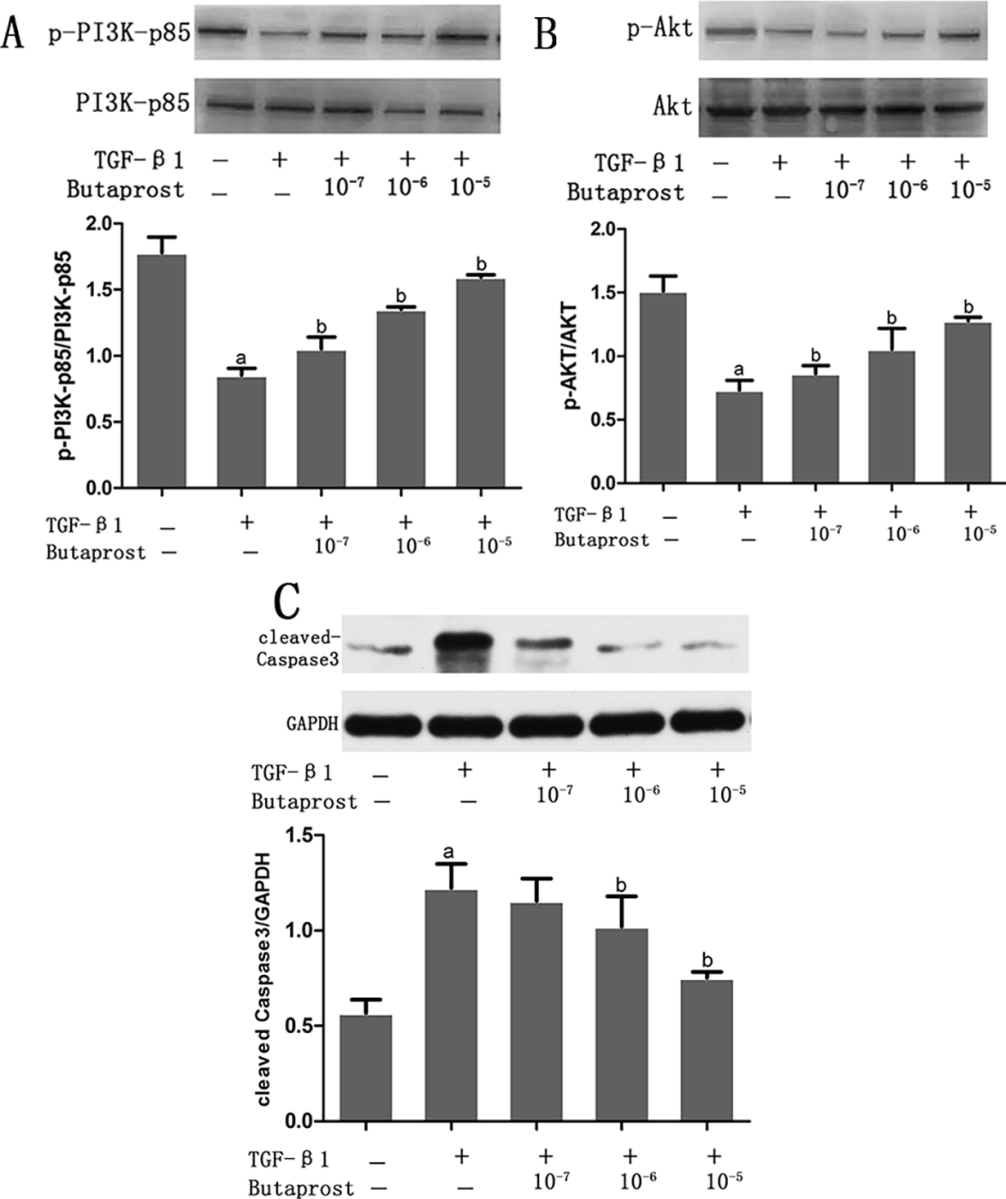
The effect of Butaprost on PI3K, Akt, and caspase3 expression in TGF-β1- induced podocytes. Podocytes were pretreated with different concentrations (10^−7^,10^−6^, or 10^-5^mol/L) of Butaprost (n = 4 each dose) for 30 min, then treated with 5 ng/ml TGF-β1 for 24 h. p-PI3K, p-Akt, and cleaved-caspase 3 were determined at the protein level.(A: p-PI3K, B: p-Akt, C: cleaved-caspase3) ^a^P<0.05 compared to the control group, ^b^P<0.05 compared to the TGF-β1 group.

**Fig 9:**
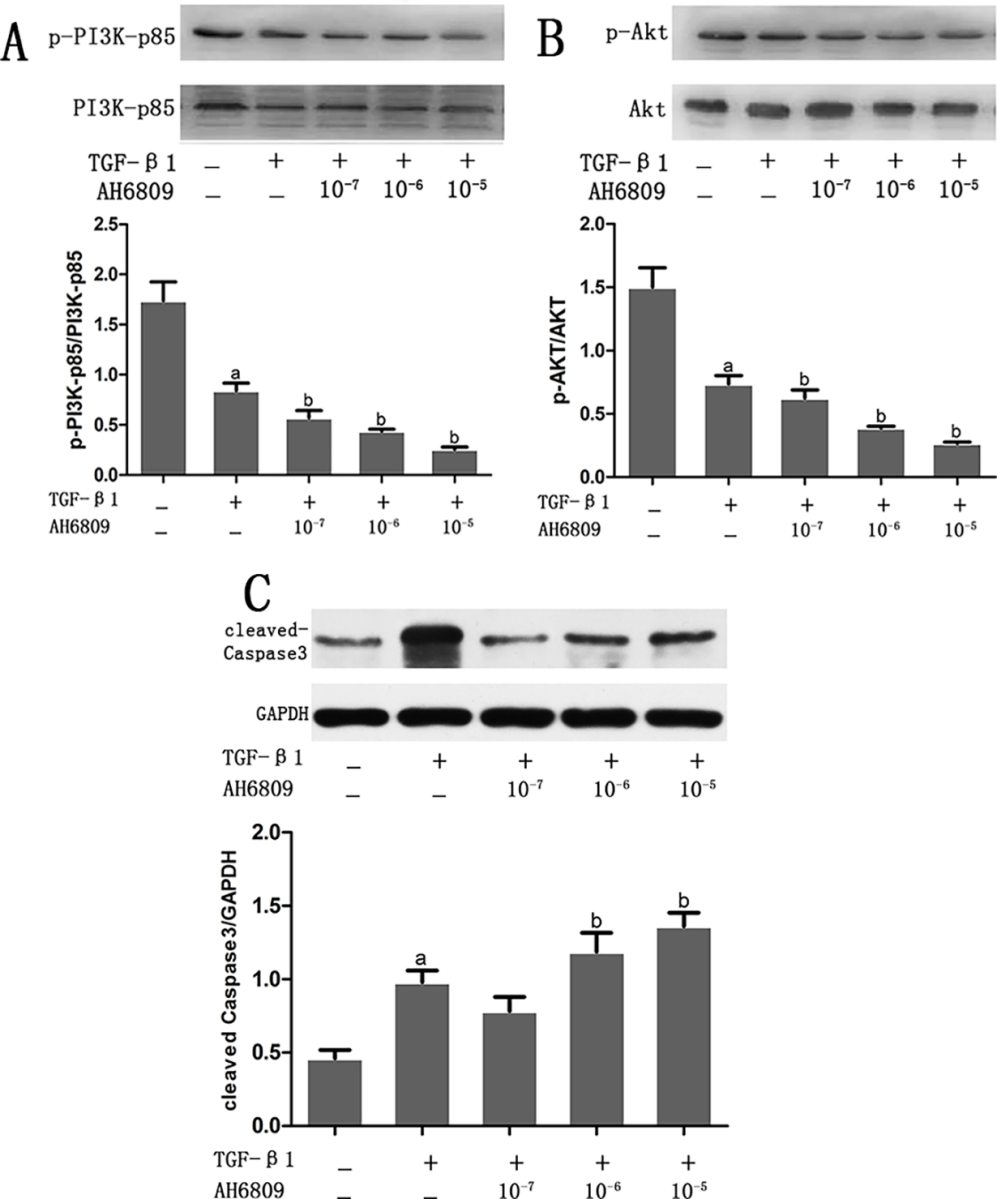
The effect of AH6809 on PI3K, Akt, and caspase3 expression in TGF-β1- induced podocytes. Podocytes were pretreated with different concentrations (10^−7^,10^−6^, or 10^-5^mol/L) of AH6809 (n = 4 each dose) for 30 min, and then were treated with 5 ng/ml TGF-β1 for 24 h. p-PI3K, p-Akt and cleaved-caspase 3 were determined at the protein level.(A: p-PI3K, B: p-Akt, C: cleaved-caspase3) ^a^P<0.05 compared to the control group, ^b^P<0.05 compared to the TGF-β1 group.

Western blot results showed expression of PI3K and Akt proteins were down regulated and activated caspase-3 protein was overexpressed in the TGF-β1 group compared with the control group (*P* < 0.05) (Figs 8 and 9). Compared with the TGF-β1 group, phosphorylated PI3K and Akt were overexpressed and activated caspase-3 protein was decreased in the Butaprost group. (Fig 8). However, in the AH6809 group, phosphorylated PI3K and Akt were down regulated and activated caspase-3 protein overexpressed, both in a time-and dose-dependent manner (*P* < 0.05). (Fig 9).

### Effect of Butaprost and AH6809 on podocytes apoptosis induced by TGF-β1

**Fig 10:**
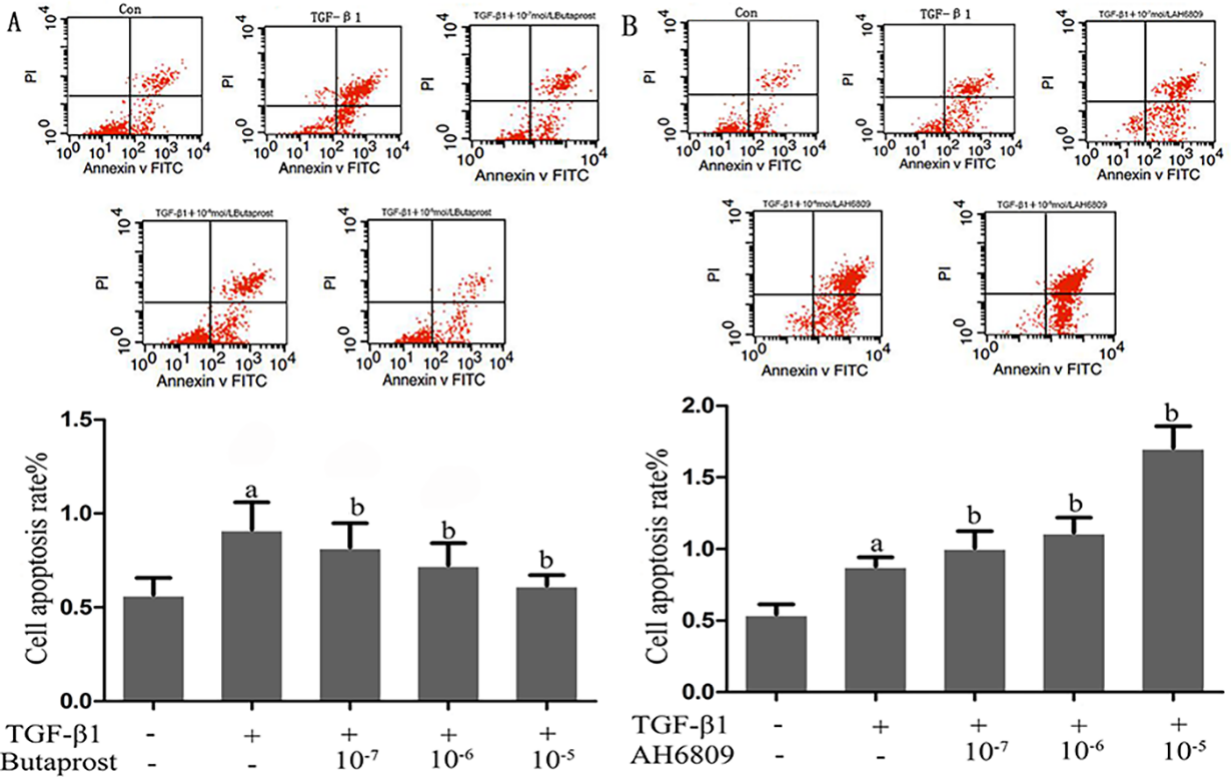
The effect of EP2 on apoptosis of TGF-β1-induced podocytes. Flow cytometry analysis were performed to determine the apoptosis of podocytes. A: Podocytes were pretreated with different concentrations (10^−7^, 10^−6^, or 10^-5^mol/L) of Butaprost (n = 4 each dose) for 30 min. B: Podocytes were pretreated with different concentrations (10^−7^, 10^−6^, or 10^-5^mol/L) of AH6809 (n = 4 each dose) for 30 min. ^a^P<0.05 compared to the control group, ^b^P<0.05 compared to the TGF-β1 group.

Flow cytometry analysis showed that podocyte apoptosis significantly increased in the TGF-β1 group compared with the control group (*P* < 0.05). Apoptosis dramatically decreased in the Butaprost group, but increased in the AH6809 group compared with TGF-β1, and both in a dose-dependent manner (*P* < 0.05). (Fig 10).

## Discussion

Various factors participate in chronic kidney diseases and end stage of renal diseases, including epithelial mesenchymal transition (EMT), TGF-β1, connective tissue growth factor (CTGF), angiotensinⅡ, and inflammation[14]. TGF-β1 is a pleiotropic cytokine that can lead to the occurrence and development of fibrosis as well as initiate and adjust a variety of pathophysiologic processes [15]. The role of TGF-β1 has been well established as a major contributor to the sclerotic glomeruli[16]. TGF-β1 over-expression in podocytes can activate p38 MAPK and caspase-3, induce podocyte apoptosis, and trigger EMT, ultimately give rise to proteinuria and glomerular sclerosis induced by podocyte detachment from the GBM when the cell phenotype is altered and cell structure and function is lost [17, 18]. In addition, TGF-β1 can activate the NF-κB pathway and signal the induction of COX2 protein, which plays a cardinal role in the pathophysiology of glomerular diseases[19]. Subsequently, PGE2 released in the kidney resulted in microtubule reorganization and foot process effacement of podocytes. PGE2 exerts various observable effects on controlling kidney functions, which are related to the distribution of these four receptor proteins[20]. Wissam H et al[21] found that PGE2 initiates a positive feedback loop in podocytes that drives p38 MAPK activity and COX-2 expression through a cAMP/AMPK dependent, and this PGE2-induced signaling network could be detrimental to podocyte health and glomerular filtration barrier integrity. In addition, PGE2 directly induces Stat3 activation in podocytes, which upregulates Stat3-derived inflammatory cytokines IL-7, IL-6, MCP1, and ICAM-1[22]. Many studies show that PGE2 leads to podocytes injury by triggering inflammation [23].

Abdallah et al first documented that TGF-β1 is able to induce cyclooxygenase protein translation in podocytes, and ultimately leads to concomitant production of PGE2 causing Akt inhibition and TAU protein hyper-phosphorylation leading to podocyte injury. We hypothesized that TGF-β1 mediates podocyte injury through other signaling pathways. Our study showed that the expression of nephrin, podocin, and CD2AP mRNA and protein in podocytes were significantly decreased after 24 h of TGF-β1 stimulation, whereas the expression of activated caspase-3 protein was significantly increased. The apoptosis rate of the TGF-β1 group was significantly higher than that of the control group. These results suggest that TGF-β1 stimulation may mediate podocyte injury by reducing the expression of SD proteins and mediating podocyte apoptosis by activating caspase-3.

Protection of podocytes is of important for glomerular diseases therapies in the clinic[24]. In our previous study, we demonstrated that the COX2/PGE2/EP cascade played an important role in TGF-β1 induced mesangial cell injury. The stimulation of the EP2 receptor inhibits the MAPK/CREB pathway and its downstream effectors, cell cycle proteins and CTGF via an increase in cAMP levels, thus contributing to inhibition of TGF-β1-induced mesangial cell injury[10].Based on these studies, we propose a hypothesis that EP2 intervention can alleviate TGF-β1-induced podocyte injury. EP2 mainly couples with Gs protein, which enhance intracellular cAMP levels and activates PKA and its downstream signaling molecules. cAMP is an important second messenger, and plays a role in cell proliferation, differentiation, and cytoskeleton formation via the PKA signaling pathway. Intracellular overexpression of cAMP could alleviate podocytes apoptosis induced by puromycin[25].Here, immortalized mouse podocyte cell lines were cultured in vitro. Then, a selective EP2 agonist, Butaprost, or an EP2 antagonist, AH6809 was added for pretreatment ahead of TGF-β1 induced injury. We found that Butaprost could significantly enhance podocyte proliferation, reduce apoptosis, and increase cAMP expression and decrease PGE2 expression. However, the opposite results were obtained after pretreatment with AH6809. The results indicated that activation of the EP2 receptor could promote podocyte proliferation and decrease apoptosis induced by TGF-β1. One possible mechanism is that the EP2 agonist could increase cAMP expression in podocytes. Thereafter, cAMP overexpression could have an anti-apoptotic effect by promoting podocyte differentiation, actin skeleton recombination and extracellular matrix accumulation. Meanwhile, cAMP overexpression inhibits expression of PGE2 through negative feedback, which further ameliorated podocytes injury induced by PGE2.

The majority of kidney diseases start in the glomerulus due to the susceptibility of the renal filtration unit to metabolic, genetic, mechanic, and immunologic damage. Genetic studies have identified essential podocyte proteins, which maintain the barrier. Of critical importance are SD proteins, such as podocin, nephrin and CD2AP, and cytoskeletal proteins such as α-actinin-4 (ACTN4). Rinschen et al provided direct and systems-level evidence that the SD and podocyte cytoskeleton are regulated targets of proteolytic modification, which is altered upon podocyte damage [26]. In the present study, we detected the expression of SD proteins after treating podocytes with EP2 agonist. Results showed that expression of nephrin, podocin and CD2AP mRNA and proteins of podocytes pretreated with Butaprost significantly increased compared with the TGF-β1 group. However, the EP2 antagonist AH6809 pretreated podocytes had decreased expression of podocyte marker proteins and exacerbation of injury, which indicated that EP2 could reinforce expression of podocyte marker proteins including nephrin, podocin, and CD2AP and promote interaction among them to maintain normal SD ultra-structure, stabilize cytoskeleton, ameliorate podocyte injury induced by TGF-β1, and maintain normal podocyte pathophysiological functions.

Furthermore, we assessed a signaling pathway EP2 was able to protect in podocytes. In addition to maintaining normal SD ultra-structure and function, nephrin and CD2AP can also participate in podocyte signal transduction by activating PI3K/Akt-dependent signal pathway via combination with PI3K. PI3K family members are second messengers in intracellular signal transduction and consist of the regulatory subunit (p85) and catalytic subunit (p110). The subtype PI3K- p85 can interact with Phosphorylated tyrosine residues and can be activated via dipolymer conformational change [27]. Zhu et al [28] found that tyrosine phosphorylation of nephrin could regulate interaction between nephrin and PI3K, then result in PI3K protein activation and down-stream signal transduction reinforcement. In addition, CD2AP is essential for TGF-β1-induced early activation of the anti-apoptotic PI3K/Akt pathway. Lack of CD2AP intensifies the activation of the pro-apoptotic p38 MAPK pathway by TGF-β1[29]. CD2AP could also facilitate PI3K/Akt signal pathway activation induced by nephrin, and ultimately protect podocytes from apoptosis[30]. The present study showed that the expressions of phosphorylated PI3K/Akt proteins were significantly decreased and activated caspase-3 protein was overexpressed in podocytes stimulated by TGF-β1. In addition, flow cytometry showed increased podocyte apoptosis. EP2 agonist Butaprost treatment could ameliorate both decreased phosphorylated PI3K/Akt proteins expression and increased activated caspase-3 protein expression. However, EP2 antagonist AH6809 treatment could further decrease PI3K/Akt proteins expression and increase apoptosis. The results may model activated EP2, which could promote the binding of phosphorylated nephrin with PI3K-p85 thereby activating the signal transduction molecule Akt, promoting podocytes proliferation and survival, and alleviating podocyte apoptosis.

In conclusion, TGF-β1 could induce podocyte injury and apoptosis by reducing expression of SD proteins. Specific activation of EP2 could ameliorate TGF-β1 induced podocyte injury by promoting cell proliferation, stabilizing cytoskeleton structure, as well as reducing apoptosis. We hypothesize that activated EP2 could enhance expression of cAMP. cAMP is not only able to inhibit PGE2 expression by negative feedback, but also promote phosphorylation of nephrin. Phosphorylated nephrin can interact with podocin and CD2AP to stabilize of podocyte cytoskeleton structure and inhibit podocyte apoptosis by activating the PI3K/Akt signal pathway. Thus, EP2 activation can exert a protective function during podocyte injury. Further studies in animal models of podocyte injury are necessary in order to evaluate in vivo the potential protective effects of EP2 receptor activation in ameliorating the disease progression.

## Supporting information

S1 File The original OD value of each group in the cck8 experiment. (XLSX)

S2 File The original data for each group in the ELISA experiment. (XLSX)
